# The effect of abstract versus concrete framing on judgments of biological and psychological bases of behavior

**DOI:** 10.1186/s41235-017-0056-5

**Published:** 2017-03-20

**Authors:** Nancy S. Kim, Samuel G. B. Johnson, Woo-kyoung Ahn, Joshua Knobe

**Affiliations:** 1 Department of Psychology, Northeastern University, 125 Nightingale Hall, 360 Huntington Avenue, Boston, MA 02115 USA; 2 Department of Psychology, Yale University, Box 208205, New Haven, CT 06520-8205 USA; 3 Department of Philosophy, Yale University, 344 College Street, New Haven, CT 06511 USA

**Keywords:** Person perception, Causal attribution, Explanation, Framing effect, Science education

## Abstract

Human behavior is frequently described both in abstract, general terms and in concrete, specific terms. We asked whether these two ways of framing equivalent behaviors shift the inferences people make about the biological and psychological bases of those behaviors. In five experiments, we manipulated whether behaviors are presented concretely (i.e. with reference to a specific person, instantiated in the particular context of that person’s life) or abstractly (i.e. with reference to a category of people or behaviors across generalized contexts). People judged concretely framed behaviors to be less biologically based and, on some dimensions, more psychologically based than the same behaviors framed in the abstract. These findings held true for both mental disorders (Experiments 1 and 2) and everyday behaviors (Experiments 4 and 5), and yielded downstream consequences for the perceived efficacy of disorder treatments (Experiment 3). Implications for science educators, students of science, and members of the lay public are discussed.

## Significance

In everyday life, we tend to frame behaviors in different ways. Sometimes we talk about behavior in general terms (e.g. some people stay calm in competitive situations; some people lose pleasure in activities that they once enjoyed). At other times, we talk about those same behaviors with reference to particular people in the context of their lives (e.g. Allen stayed calm during his figure-skating competition; Dan no longer takes pleasures in long country drives). The question is whether these different kinds of descriptions matter; that is, does framing affect the inferences we make about those behaviors? Although these abstract and concrete descriptions seem to essentially depict the same behaviors, we found that the two levels of description lead to different judgments about how to explain the behavior. Across five studies, participants favored biological explanations (e.g. brain chemistry; genetics) more for abstract descriptions than for concrete cases and they favored some psychological explanations (e.g. intentions; emotions) more for concrete cases than for abstract descriptions. These shifts in people’s preferences occurred both for ordinary behaviors (e.g. Allen’s calm behavior) and mental disorder symptoms (e.g. delusions). As neuroscience and genetics research have increasingly been capturing the public’s attention, we argue that these results have important implications for science education and for public health communication.

## Background

In the real world, unusual human behaviors (e.g. the symptoms of schizophrenia) are often described at one of two distinct levels of abstraction. At one level, behaviors are described in the abstract, as generalized across individuals. For example, when we google the word “schizophrenia,” the websites that immediately come up—from the National Institute of Mental Health, Mental Health America, National Alliance for the Mentally Ill, Wikipedia, schizophrenia.com, and so on—provide abstract descriptions of schizophrenia and its symptoms (e.g. delusions). Abstract descriptions are also found when we search through an encyclopedia, dictionary, or medical handbook. At another level, we also talk about specific instances of the same behaviors (e.g. a woman who strongly believes that the next-door neighbor is her husband when in fact they have not met). One might learn about the concrete symptoms of schizophrenia via the depiction of a particular person in a film (e.g. *A Beautiful Mind*; Howard, 2001), book (e.g. *I Know This Much Is True*; Lamb, 2008), or magazine article about an individual. One might also learn by observing such symptoms first-hand in a friend or family member, or hear about other specific cases by word of mouth.

Our central question is whether there is any effect of the level of abstraction at which the behaviors are described. Previous studies showed that concrete examples affect judgments more strongly than abstract descriptions do, because concrete examples are more salient, memorable, or convincing (e.g. Borgida & Nisbett, 1977; Jenni & Loewenstein, 1997; see also Semin & Fiedler, 1991 for different ways of construing abstract versus concrete descriptions). In the current work, we ask whether learning about behaviors in the abstract versus from a concrete instance significantly shifts the kinds of inferences laypeople then draw about the behavior. In particular, we approach this question in terms of two different types of explanations for behaviors that are pervasive in lay discourse (as well as scientific): psychological and biological explanations.

People often see human behaviors being explained in terms of psychological constructs. For instance, one might explain that a person has been feeling depressed because she is under too much unrelenting stress at work. More recently, as the field of neuroscience has rapidly progressed, people have also become familiar with biological explanations for behaviors (O’Connor & Joffe, 2013). For example, one could also explain that a person has been feeling depressed due to a neurochemical imbalance. As we will see in the next section, there are multiple possible ways in which the level of abstraction at which behaviors are depicted (i.e. abstractly or concretely) affects which types of explanations (i.e. psychological and biological) laypeople believe to be more plausible.

### Relations between abstract versus concrete framing and biological versus psychological explanations

We hypothesize that laypeople are relatively accepting of biological explanations of behaviors in the abstract, but are more reluctant to accept such explanations for the behavior of concrete individuals. For instance, when contemplating generalized anxiety disorder, laypeople may be generally accepting of neurological or genetic explanations. Yet, when confronted with a particular concrete individual with generalized anxiety disorder displaying specific anxiety symptoms, people may be less inclined to endorse biological explanations and instead explain that individual’s symptoms as intentional or controllable. Such findings could have considerable implications for public health, given that judgments of intentionality or controllability are critical in driving stigma towards abnormal behaviors and the stigmatizing attitudes of others have enormous impact on treatment seeking, treatment avoidance, and benefits from treatment (e.g. Pescosolido, Martin, Lang, & Olafsdottir, 2008).

A recent study found empirical support for a similar hypothesis in practicing mental health clinicians’ inferences about biological and psychological bases of symptoms of mental disorders (Kim, Ahn, Johnson, & Knobe, 2016). We found that hallmark symptoms of disorders described in the abstract led expert clinicians to endorse their biological basis more strongly, and their psychological basis less strongly, than when the same symptoms were described concretely (i.e. in terms of individual cases). For instance, clinicians judged a disorder “characterized by loss of pleasure” involving “feeling a substantially diminished interest in most activities, including activities found enjoyable in the past” to be more biologically caused than Dan’s problems of no longer showing “interest in most activities, no longer taking pleasure in golfing or long country drives, even though these used to be some of his very favorite weekend activities.” In addition, clinicians were more likely to endorse the effectiveness of medication when they received the abstract description than when they received the concrete description, even though a pretest verified that the two descriptions were judged to be essentially equivalent.

However, it is unclear whether these findings are generalizable outside the population of clinicians and the domain of mental health. It is possible that clinicians are a special case, because in their intensive initial training and continuing education, clinicians generally learn biological explanations for behavior in abstract form. Much like laypeople, clinicians frequently encounter psychological explanations in their ordinary concrete interactions, and in their training, clinicians are exposed to psychological evaluations of individual case studies in clinical practice and through client case formulations (Eells, Kendjelic, & Lucas, 1998). Importantly, however, clinicians are also exposed throughout their training to biological explanations through more abstract discussions in textbooks and research articles (e.g. describing new evidence for the neurochemical bases of schizophrenia). By contrast, laypeople have a great deal of concrete experience with psychological explanation, but compared to clinicians, they typically have far less exposure to abstract discussions of biological explanation. One might therefore predict that laypeople would not show the effect observed among trained clinicians.

One might even further argue that because psychological states (e.g. intentions, stress) are not tangible in nature, laypeople may actually see them as being more abstract than biological states, which refer to tangible things such as the physical brain. Furthermore, from a reductionist viewpoint, biological explanations would be considered lower level explanations for behaviors than psychological explanations for the same behaviors. Within the hierarchy of levels of explanation, psychological explanations are more abstract than biological ones, being relatively lacking in concrete, physically grounded detail (e.g. Dennett, 1971). As a result, laypeople might find abstractly framed stimuli to be more compatible with psychological construals of behaviors than with biological construals.

Still, there are some potential reasons to expect that the framing effects previously obtained with practicing clinicians may turn out to reflect a broader, more general phenomenon. First, in linguistics, a distinction is made between generic statements (i.e. generalizations that are made about entire categories of people or things, such as “girls wear pink”) and non-generic statements (i.e. statements that are not generic, such as descriptions of specific individuals like “Mary wears pink;” see Cimpian & Erickson, 2012). Studies suggest that laypeople prefer to explain generics in terms of inherent features (e.g. pink is delicate and girls are hardwired to be attracted to it) rather than external features (e.g. it is merely a societal convention for girls to wear pink; Cimpian & Salomon, 2014). In addition, biological properties are perceived to be more permanent, immutable, and timeless than psychological properties (e.g. Dar-Nimrod & Heine, 2011; Haslam, Bastian, & Bissett, 2004). For instance, the more that people with depression attribute their symptoms to biological factors such as brain abnormalities or genes, the more pessimistic they are about recovery (Lebowitz, Ahn, & Nolen-Hoeksema, 2013). Taken together, findings such as these suggest that biological explanations may seem more compatible with abstract framing, which describes timeless patterns, than with concrete framing, which describes transient events. Second, psychological explanations may be more salient to laypeople when a behavior is described concretely than when it is described in the abstract. This idea is supported by past work on people’s intuitions about free will. When laypeople are told in the abstract about a universe in which everything is fully determined, they tend to say that no agent in this universe can be morally responsible for his or her behavior, but when people are told about one specific agent in the same deterministic universe, they tend to say that this specific agent actually is morally responsible (Nichols & Knobe, 2007). This effect arises because people reading a concrete case are more inclined than are people reading about an abstract case to think that the agent’s behavior was best explained by his or her psychological states (Murray & Nahmias, 2014). Thus, concrete descriptions of individual agents performing specific actions may make psychological states (e.g. intentions, feelings) salient in a way that more abstract descriptions do not (Nichols & Knobe, 2007; Sinnott-Armstrong, 2008).

### Overview of experiments

The main goal of the current experiments was to examine whether laypeople’s biological (and psychological) judgments are affected by the abstract versus concrete framing of behaviors and, if so, in what direction judgments are affected. We tested these hypotheses by measuring people’s endorsements of various biological and psychological explanations for behavior, across a range of equivalent abstract and concrete cases.

There are many ways to manipulate the abstractness of behavior descriptions and many ways to determine which levels of abstractness should be of primary interest. We modeled our experimental manipulations on a distinction frequently encountered in the real world. The abstract version simulates general descriptions of behaviors; that is, these descriptions make reference to people exhibiting the behavior in general and describes behaviors in the abstract (e.g. coming up with strange beliefs that are contrary to fact and that persist strongly despite having no evidence to support them), as in nosologies such as the *Diagnostic and Statistical Manual of Mental Disorders* (*DSM-5*, 5th ed., American Psychiatric Association, 2013). The concrete version makes reference to a particular person and describes behaviors as specifically instantiated in the context of that person’s life (e.g. Jenny has developed the strong belief that the man living next door is her husband), as in casebook training manuals for learning nosologies such as *DSM-5 Clinical Cases* (Barnhill, 2013). This way of manipulating abstractness is the same as that deployed in Kim et al.’s (2016) study with clinicians, allowing us to compare the current results (Studies 1, 2, and 3) with those from experts in the domain. Unlike in Kim et al.’s (2016) study, however, we also used stimuli that are not symptoms of mental disorders because of the current focus on laypeople rather than clinicians (Studies 4 and 5). For example, participants in our studies might read about either how some people stay calm during competitive situations (abstract description described generally) or how Allen stayed calm during a figure-skating competition (concrete, individual case described within the specific context of that person’s life).

Our prediction is that biological explanations are more strongly endorsed in the abstract than in the concrete, and that psychological explanations of behavior are more strongly endorsed in concrete cases than in the abstract. That is, we would expect laypeople to think that brain chemistry, neural structure, and so on are better explanations of calm performance in general than of Allen’s calm performance in particular. Conversely, we predict that explanations attributing calm performance to intentions or emotions would be endorsed more for Allen’s calm performance than for calm performance in general.

We tested these predictions across five experiments. Experiments 1 and 2 compared laypeople’s judgments of the biological (and psychological) bases of various mental disorders. Each disorder was described in a concretely or abstractly framed vignette, judged by pretest participants to be essentially equivalent. Experiment 3 tested whether these inferences have downstream consequences for how people would choose to intervene on disordered behavior—by using medication or by using psychotherapy. Finally, Experiments 4 and 5 extended these results beyond the domain of mental disorders, examining lay judgments for behaviors that are uncommon (and hence in need of explanation) but not the result of mental disorders.

## Experiment 1

Experiment 1 tested whether laypeople’s causal attributions for disordered behavior are shifted by abstract versus concrete framing. Although clinicians tend to view behaviors as more biologically based in the abstract than in the concrete, and more psychologically based in the concrete than in the abstract (Kim et al., 2016), it is unclear whether this effect is largely induced by clinical training and practice, or whether it would also extend to laypeople.

This question has considerable practical import, because laypeople’s attributions for mental disorders influence many outcomes of real-world importance. More biological attributions for disordered behavior reduce judgments of blame for symptoms (e.g. Corrigan & Watson, 2004), but can increase essentialism (Haslam & Ernst, 2002), leading to greater pessimism about recovery (e.g. Dar-Nimrod & Heine, 2011; Lebowitz et al., 2013). Furthermore, biological attributions for symptoms are associated with the belief that medication is a more effective treatment than psychotherapy (e.g. Iselin & Addis, 2003; Luk & Bond, 1992; Yopchick & Kim, 2009). The potential for abstract versus concrete framing to affect such construals is a pressing issue in need of examination, given that people frequently encounter both abstract descriptions of disorder symptoms (e.g. on WebMD) and concrete cases (e.g. their loved ones who have disorder symptoms).

In addition, we probed the boundaries of this framing effect by asking participants about various types of biological and psychological attributions. In previous work (Kim et al., 2016), clinicians were asked to what extent the behaviors are “biologically based” or “psychologically based” in general, rather than about specific types of biological and psychological causes. Yet, there are many different kinds of both biological explanations (e.g. brain structure, genetics) and psychological explanations (e.g. in terms of cognition, emotion, or intentions). To what extent would shifts in attributions generalize across these types of biological and psychological causation? We tested these questions in Experiment 1 by asking participants to make judgments about several different types of biological and psychological causation for disordered behavior.

### Method

#### Participants

Fifty-one participants were recruited via Amazon Mechanical Turk (see Buhrmester, Kwang, & Gosling, 2011). Eight were excluded from analysis (*N* = 3 due to taking similar studies in the past and *N* = 5 due to random responses on filler items).

#### Materials and pretest

We selected six items, each a hallmark symptom of a well-known disorder in the *DSM-IV-TR* (American Psychiatric Association, 2000).^1^ For each item, we wrote an abstract version approximating the level of description in the *DSM-IV-TR* (American Psychiatric Association, 2000), and a corresponding concrete version detailing behaviors exhibited by a specific person (approximating the level of description in the *DSM-IV-TR Casebook*; Spitzer, Gibbon, Skodol, Williams, & First, 2002). The two versions were roughly equated for length (see Table 1).

**Table 1:** Stimuli for Experiments 1–3

Item	Text version	
	Concrete	Abstract
1. Delusional thoughts and behaviors	Jenny has developed the strong belief that the man living next door is her husband; she sometimes follows him when he is driving and she sends hate mail to his actual wife, though she has never actually met either of them in person.	This disorder is characterized by delusional thoughts and behaviors; it involves coming up with strange beliefs that are contrary to fact and that persist strongly, influencing daily behaviors, despite having no evidence to support them.
2. Manic beliefs and behaviors	Eric effusively talks about his dozens of highly unrealistic business ideas, which he thinks are guaranteed to make him millions of dollars; he erroneously believes that he is irresistibly attractive to much younger women and is oblivious to their rejections.	This disorder is characterized by manic beliefs and behaviors; it involves holding extremely positive self-views, which are often completely unfounded in reality, and often talking excitedly about all of these beliefs, despite the fact that they are untrue.
3. Loss of pleasure	Dan no longer shows interest in most activities, no longer taking pleasure in golfing or long country drives, even though these used to be some of his very favorite weekend activities.	This disorder is characterized by loss of pleasure; it involves feeling a substantially diminished interest in most activities, including activities found enjoyable in the past.
4. Repetitive, compulsive behaviors	Sarah locks each of her windows three times whenever she leaves her house in order to prevent a burglary, she uses a new bar of soap every time she washes her hands, and she runs a virus scan on her computer every hour, even when her computer is disconnected from the Internet.	This disorder is characterized by repetitive behaviors; it involves feeling compelled to repeatedly engage in behaviors aimed at preventing some dreaded event, even though these behaviors are not a realistic means for preventing what they are intended to prevent.
5. Feelings of worthlessness/guilt	Chris believes that he is incompetent at his job, despite excellent performance evaluations, and blames himself for his company’s recent financial losses that were actually caused by uncontrollable circumstances; when a busy co-worker passes by him without engaging in a lengthy conversation, he thinks it is because he is inherently unlikeable.	This disorder is characterized by feelings of worthlessness, with unrealistically negative self-evaluations; it involves an exaggerated sense of guilt and personal responsibility for negative occurrences and interpreting neutral, day-to-day events as evidence of personal defects, even though these occurrences are not realistic reflections of poor character.
6. Recurrent nightmares	Mike has nightmares almost every night; he often dreams that he is a passenger on an airplane that is out of control and about to crash, or that he has been kidnapped by a serial killer who is planning to torture him.	This disorder is characterized by frequent nightmares; it involves having terrifying dreams more nights than not, which often portray threats to physical safety and may involve life-threatening situations.

Because we are testing the effect of abstract versus concrete framing of the same behavior, we recruited a separate group of 40 participants from Amazon Mechanical Turk to complete a pretest, measuring whether the abstract and concrete version of each behavior correspond to each other. Each behavior was shown on a separate page and the two versions of each behavior, abstract and concrete, were presented side by side on the page. As an attention check, two filler items not designed to be equivalent were also included. Four participants failed this check. Of the remaining 36 pretest participants, 15 judged whether the abstract version was “a good abstract description” of the concrete version on a scale of 1–9 (where 1 = a very poor description; 9 = a very good description), while 21 judged whether the concrete version was “a good example” of the abstract version on a scale of 1–9 (where 1 = a very poor example; 9 = a very good example). The mean rating for the “good abstract description” question was 7.97 (*SD* = 0.30); the mean rating for the “good example” question was 8.21 (*SD* = 0.29). Mean ratings by item were all at least 7.60. Thus, these pretest results verified that each pair of abstract and concrete versions is fairly equivalent.

For the main experiment, we added abstract and concrete versions of two filler items (i.e. having an unusually large brain size; having a brain tumor) to allow for attention and comprehension checks. If participants paid attention to the task, these filler items should receive very high ratings on biological questions and very low ratings on psychological questions. Five participants who did not show this pattern for the two filler items (i.e. giving responses at least two standard deviations below the mean on the biological questions [the average of Q1–3 below] or two standard deviations above the mean on one of the sets of psychological questions [the average of Q4–6 or Q7–9 below]) were excluded from the final data analyses.

For the main experiment, nine questions were developed to measure people’s judgments of the biological and psychological bases of behaviors. Three biological questions were designed to probe beliefs about biological causes of behaviors:

- Q1. Do you think [their/her/his] brain chemistry is different from that of people who [are not like this/do not do this]?
- Q2. Do you think [their/her/his] brain structures are different from those of people who [are not like this/do not do this]?
- Q3. Do you think there is a genetic basis for this?

Because naïve biology is likely to be limited, only three questions could be developed (e.g. additional questions regarding neuromodulators, etc., would not be meaningful if laypeople did not have a strong intuitive understanding of them). In contrast, because the existing literature suggests that naïve psychology encompasses a number of aspects of behavior (e.g. Malle & Knobe, 1997; Waytz, Gray, Epley, & Wegner, 2010), limiting the possible psychological questions to three to match the number of biological questions would unnecessarily restrict the scope of the findings. Six questions were therefore gathered to probe beliefs in psychological causes of behaviors:

- Q4. Do you think this is caused by cognitive factors (e.g. [their/her/his] beliefs, knowledge, intelligence, or thinking style)?
- Q5. Do you think this is caused by [their/her/his] emotions and desires?
- Q6. Do you think this is caused by [their/her/his] [personalities/personality]?
- Q7. Do you think [they are/she is/he is] intentionally [like this/doing this]?
- Q8. Do you think [they/she/he] should be [held responsible for/given credit for] [being like this/doing this]?
- Q9. Do you think the causes of this are under [their/her/his] control?

Q4, Q5, and Q6 (Psychological Set 1) were derived from tables of contents of Introductory Psychology textbooks as factors that are frequently addressed in the study of individual differences. Q7, Q8, and Q9 (Psychological Set 2) were derived from questions measuring beliefs about agency (e.g. Weiner, 1995, 2001).

Participants responded to these questions on scales of 1–7 (where 1 = not at all; 7 = definitely). For each version of each behavior, the nine questions were presented in randomized order across participants and across items. For each item, participants completed the nine explanation judgments on the same screen, with each item presented on a separate screen.

#### Procedure and design

All experiments were programmed using the online survey software *Qualtrics* (Qualtrics Labs, Inc., 2005). After reading a general overview of the task, each participant completed two blocks of items. Each block began with a filler item, followed by the six disorders listed in Table 1, with half of the disorders in the abstract version and half in the concrete version, presented in a random order. The second block contained the abstract versions of the concrete items from the first block, and the concrete versions of the abstract items from the first block. That is, participants rated both the abstract and concrete versions of each item, with the two versions in separate halves of the experiment in a counterbalanced order. From the participants’ perspective, there was no obvious marking for filler items or switching between blocks. Upon completing all items, participants completed a dualism scale (Stanovich, 1989).

To summarize, the experiment incorporated a 2 (abstract or concrete) × 2 (psychological attributions or biological attributions) within-subjects design.

### Results

We first computed a biological score for each item by averaging each participant’s responses to the three biological measures (Cronbach’s α = 0.97, calculated by item), and a psychological score for each item by averaging each participant’s responses to the six psychological measures (α = 0.97).

**Fig. 1:**
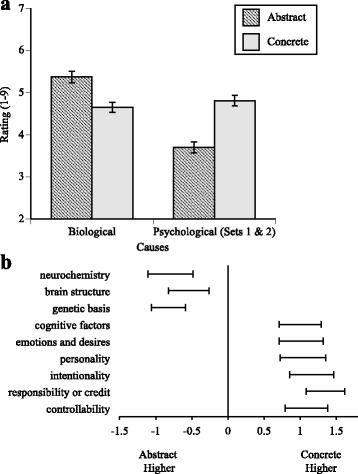
Results of Experiment 1. **a** Judgments of the biological and psychological bases of disordered behaviors rated within-subject; bars depict Cousineau–Morey standard errors (Cousineau, 2005; Morey, 2008). **b** The 95% confidence intervals of the difference scores (concrete minus abstract) for ratings on the nine dependent measures. “Biological Causes” in (**a**) are the averaged ratings of the first three dependent measures listed in (**b**), and “Psychological (Sets 1 & 2) Causes” are the averaged ratings of the last six dependent measures listed in (**b**)

We predicted that biological attributions would be greater for the abstract version than for the concrete version and that psychological attributions would be greater for the concrete version than for the abstract version. To test this, we conducted a 2 (concrete or abstract) × 2 (biological or psychological) repeated measures ANOVA on each participant’s mean across items. This analysis revealed the predicted interaction, *F*(1,42) = 95.68, *p* < 0.001, η_p_^2^ = 0.70, as shown in Fig. 1a. Biological attributions were higher for the abstract versions (*M* = 5.37, *SD* = 1.23) than for the concrete versions (*M* = 4.65, *SD* = 1.16), *t*(42) = −6.32, *p* < 0.001, *d* = −0.96, while psychological attributions were higher for the concrete versions (*M* = 4.80, *SD* = 0.89) than for the abstract versions (*M* = 3.70, *SD* = 0.99), *t*(38) = 10.85, *p* < 0.001, *d* = 1.65.

Figure 1b shows the 95% confidence intervals of the difference scores (concrete minus abstract) for each of the nine component measures. Each measure yielded a difference score that was significantly negative for all three biological measures, indicating a stronger preference for biological explanations in the abstract, and significantly positive for all six psychological measures, indicating a stronger preference for psychological explanations in the concrete.

The interaction effect also held up in a by-item analysis, using each item’s mean score across participants. A 2 (abstract or concrete) × 2 (biological or psychological) repeated measures ANOVA on these scores revealed a significant interaction, *F*(1,5) = 17.32, *p* = 0.009, η_p_^2^ = 0.78. Biological attributions were higher for the abstract versions (*M* = 5.37, *SD* = 0.30) than for the concrete versions (*M* = 4.65, *SD* = 0.89), *t*(5) = −2.58, *p* = 0.049, *d* = −1.05, while psychological attributions were higher for the concrete versions (*M* = 4.80, *SD* = 0.95) than for the abstract versions (*M* = 3.70, *SD* = 0.44), *t*(5) = 5.04, *p* = 0.004, *d* = 2.06.

### Discussion

Experiment 1 found that biological attributions were higher for abstract than concrete descriptions and psychological attributions were higher for concrete than abstract descriptions for the same behaviors. Remarkably, although neither the abstract nor the concrete version explicitly mentioned anything about the causes of the behaviors, attributions were strongly affected by the framing manipulation. Thus, not only expert clinicians (Kim et al., 2016), but also laypeople, show an effect of framing on their causal attributions for behavior. Furthermore, the effect occurred robustly across all measures we used of psychological and biological attributions, suggesting that it is quite broad.

## Experiment 2

In Experiment 1, each participant made both biological and psychological attributions. This design enabled us to demonstrate shifts within the same individual, but it is possible that participants may have felt experimenter demand to rate the biological and psychological questions in opposing directions. Experiment 2 therefore aimed to replicate the finding using a between-subjects design; that is, by having participants make only biological or only psychological judgments.

### Method

A total of 124 participants were recruited via Amazon Mechanical Turk, of whom nine were excluded (*N* = 2 due to taking similar studies in the past and *N* = 7 due to random responses on filler items).

The stimulus materials were the same as in Experiment 1. Unlike in Experiment 1, the nine questions were grouped into three sets: Biological (Q1, Q2, and Q3 as described in Experiment 1), Psychological Set 1 (Q4, Q5, and Q6), and Psychological Set 2 (Q7, Q8, and Q9). Each participant received only one of the three groups of questions (*N* = 41 for Biological, *N* = 38 for Psychological Set 1, *N* = 36 for Psychological Set 2). The six psychological questions were split into two groups to equate the total number of questions received across all participants. Sample sizes were determined by power analyses on the data from Experiment 1, with 95% power subject to a minimum of 40 participants per condition (prior to excluding random responders and repeat participants).

### Results and discussion

**Fig. 2:**
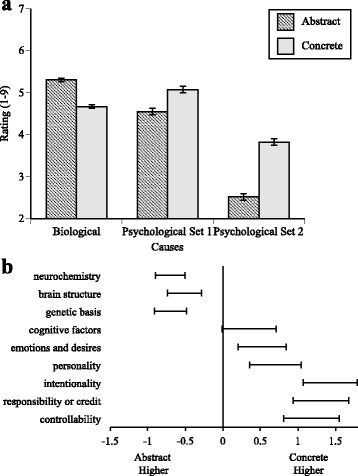
Results of Experiment 2. **a** Judgments of the biological and psychological bases of disordered behaviors rated between-subject; bars depict Cousineau–Morey standard errors (Cousineau, 2005; Morey, 2008). **b** The 95% confidence intervals of the difference scores (concrete minus abstract) for ratings on the nine dependent measures. “Biological Causes” in (**a**) are the averaged ratings of the first three dependent measures listed in (**b**), “Psychological Set 1 Causes” the second three, and “Psychological Set 2 Causes” the last three

We conducted a 2 × 3 mixed-model ANOVA on each participant’s mean across items, with framing (concrete or abstract) as a within-subjects factor and attribution type (Biological, Psychological Set 1, or Psychological Set 2) as a between-subjects factor. This analysis revealed the predicted interaction, *F*(2,112) = 54.83, *p* < 0.001, η_p_^2^ = 0.50, as shown in Fig. 2a. Biological attributions were higher for the abstract (*M* = 5.31, *SD* = 1.20) than for the concrete versions (*M* = 4.67, *SD* = 1.25), *t*(40) = −7.47, *p* < 0.001, *d* = −1.67. Conversely, psychological attributions were higher for the concrete than for the abstract versions, both for Psychological Set 1 (*M* = 5.08, *SD* = 1.51 vs. *M* = 4.55, *SD* = 1.96), *t*(37) = 3.44, *p* = 0.001, *d* = 0.56, and for Psychological Set 2 (*M* = 3.83, *SD* = 1.21 vs. *M* = 2.52, *SD* = 1.17), *t*(35) = 8.36, *p* < 0.001, *d* = 1.38. As shown in Fig. 2b, the difference scores (concrete minus abstract) were significant in the predicted direction for eight of the nine measures (*p* < 0.05, two-tailed; cognitive factors reached marginal significance in the predicted direction, *p* < 0.10).

The interaction effect also held up in a by-item analysis. A 2 (abstract or concrete) × 2 (psychological or biological) repeated measures ANOVA on the item means revealed a significant interaction, *F*(1,5) = 22.51, *p* = 0.005, η_p_^2^ = 0.15. Biological attributions were higher for the abstract versions (*M* = 5.31, *SD* = 0.26) than for the concrete versions (*M* = 4.67, *SD* = 0.64), *t*(5) = −3.04, *p* = 0.029, *d* = −1.24, while psychological attributions were significantly higher for the concrete versions (*M* = 4.45, *SD* = 0.84) than for the abstract versions (*M* = 3.54, *SD* = 0.28), *t*(5) = 3.90, *p* = 0.011, *d* = 1.59.

These results show that the strong shifts in attribution shown in Experiment 1 cannot have occurred due to demand to inversely rate biological and psychological causes. Rather, these shifts occur independently, reflecting both a stronger belief in biological causation in the abstract and a stronger belief in psychological causation in the concrete.

## Experiment 3

In Experiment 3, we tested whether the effect of abstract versus concrete framing on biological versus psychological attributions might have a downstream effect on the perceived efficacy of treatments for mental disorders. Such a finding would have implications both for psychiatric intervention and for public health, since perceived treatment efficacy can influence actual treatment efficacy (Meyer et al., 2002).

People believe that medication is more effective for disorders that they perceive to be biologically based and that psychotherapy is more effective for those they perceive as psychologically based (e.g. Iselin & Addis, 2003; Luk & Bond, 1992; Yopchick & Kim, 2009). We therefore predicted that medication would be seen as more effective in treating symptoms described abstractly rather than concretely, since abstract descriptions were more compatible with biological explanations (Experiments 1 and 2). Put differently, making an effect (e.g. a mental disorder) appear to be more biologically caused (e.g. by neurotransmitter imbalances) should make biological interventions on that causal system (e.g. medication) appear more effective. In contrast, since concrete framing makes psychological explanations more available, psychological interventions (e.g. psychotherapy) should appear more effective with concrete rather than abstract framing.

### Method

We recruited 40 participants from Amazon Mechanical Turk. Participants made judgments about the abstract and concrete versions of the same items used in Experiments 1 and 2. However, rather than judging explanations, they rated the extent to which they believed psychotherapy would be an effective treatment and the extent to which they believed medication would be an effective treatment, on separate scales from 1 (“not at all”) to 9 (“completely”). Participants were told that psychotherapy refers to “treatment by psychological means, involving repeated verbal interactions between a clinician and a client,” and that medication refers to “treatment by psychiatric, psychoactive, or psychotropic drugs.” These judgments were always made on the same page and their order was counterbalanced so that some participants always made medication judgments first and other participants always made psychotherapy judgments first. The abstract versus concrete framing was a within-subject factor with the order of the items counterbalanced as in Experiment 1, so that the abstract and concrete versions of the same item would appear in separate halves of the experiment.

### Results and discussion

**Fig. 3:**
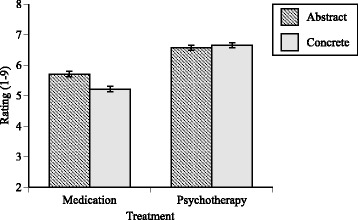
Mean judgments of medication and psychotherapy treatment efficacy in Experiment 3. Bars depict Cousineau–Morey standard errors (Cousineau, 2005; Morey, 2008)

We conducted a 2 (concrete or abstract) × 2 (medication or psychotherapy) repeated-measures ANOVA on individual participants’ means across items. This analysis revealed the predicted interaction, *F*(1,39) = 9.61, *p* = 0.004, η_p_^2^ = 0.20, as shown in Fig. 3. Medication was judged more effective when the disorder was framed abstractly (*M* = 5.71; *SD* = 1.64) rather than concretely (*M* = 5.22; *SD* = 1.60), *t*(39) = 3.70; *p* = 0.001; *d* = 0.58. However, judgments of the effectiveness of psychotherapy did not reliably differ between the abstract (*M* = 6.57; *SD* = 1.18) and concrete versions (*M* = 6.66; *SD* = 1.13), *t*(39) = 0.79, *p* = 0.43, *d* = 0.13.

When behaviors are described more abstractly, and biological explanations thereby seem more plausible (as shown in Experiments 1 and 2), the current results suggest that people come to believe that biological interventions on that causal system are more likely to influence those behaviors. These results generalize the effect of abstract and concrete framing on biological attributions to a new measure. That said, it is difficult to say whether or not the effect on treatment decisions is statistically mediated by attributions, since the effect was found for medication but not for psychotherapy. A test for mediation would require a design that measured both attributions and treatments simultaneously.

Why did the effect not extend to psychotherapy efficacy judgments? Although it is possible that this occurred because the effect of abstract/concrete framing on psychological explanations is less stable than the effect on biological explanations, we think this is not the most likely explanation. The abstractness manipulation was sufficient to find robust differences for both psychological and biological explanations in Experiments 1 and 2 and this same manipulation was used here in Experiment 3. Instead, the null effect on psychotherapy judgments is likely the result of a ceiling effect: Participants’ judgments for the psychotherapy items were between 6.5 and 7 on a nine-point scale, which may be at ceiling given people’s moderate perceptions of the degree to which psychotherapy has the potential to be effective (Jorm, 2012; Ten Have et al., 2010). In contrast, people know much less about psychotropic medications (Jorm, 2012); thus, for medication judgments they may rely more on their perceptions of the biological basis of the items, as shifted by the framing effect demonstrated in Experiment 3.

## Experiment 4

Experiments 1–3 showed that biological and psychological attributions shift depending on abstract versus concrete framing not only for clinicians (as shown in Kim et al., 2016), but for laypeople as well, and across a wide range of specific psychological and biological causes. However, these experiments leave unanswered the question of whether these attribution shifts would also occur across a wider range of human behaviors. Mental disorders may be something of a special case, because both clinicians and laypeople are accustomed to hearing both psychological and biological levels of explanation for disordered behaviors. Experiments 4 and 5 tested whether such shifts would also occur for behaviors which are more closely within the range of familiar human experience, but which are somewhat out of the ordinary and hence seem in need of an explanation.

### Method

#### Participants

Forty-nine lay participants were recruited via Amazon Mechanical Turk, of whom ten were excluded (*N* = 2 due to taking similar studies in the past and *N* = 8 due to random responses on filler items).

#### Materials and pretest

We picked eight everyday behaviors, including both positively and negatively valenced behaviors. All of these behaviors were realistic and required some explanation (e.g. having difficulty focusing on tasks for a long time; staying calm during a competitive situation; see Table 2 for a list of all stimuli). To show that the effect arises when people are thinking about everyday behaviors, we avoided highly rare behaviors, such as behaviors that were extremely positive (e.g. memorizing 100-digit matrices on a single viewing) or extremely negative (e.g. committing serial murder). In addition, to circumvent ceiling or floor effects, we avoided using behaviors for the main test items that would likely be perceived as very strongly biologically caused (e.g. breathing).

**Table 2:** Stimuli for Experiments 4 and 5

Behavior	Text version	
	Concrete	Abstract
1. Having extra-marital affairs	Douglas has been regularly sleeping with his ex-girlfriend at a local hotel; he has created an elaborate lie to tell his wife, claiming that he has to spend evenings and weekends away from the house doing extra work for his unreasonable boss.	Some men have extra-marital affairs; they have an ongoing sexual relationship with someone other than their spouse, typically without their spouse’s knowledge, and they frequently engage in deceptive behaviors to cover up these actions.
2. Having a great memory for names	Denise memorized the names of all of the students in her 85-person lecture course within the first couple of class meetings and she spent only a little extra time outside of class reviewing their names and photographs.	Some people have a great memory for names; they can learn to match a large number of names to faces under conditions of limited time, all without seeming to undergo an extraordinary amount of mental effort.
3. Being nervous in social settings	Cheryl gets nervous at all of the company dinners and parties she is expected to attend with her colleagues; she worries about whether she sounds intelligent and whether her dress, hair, and makeup look right.	Some people are nervous in social settings; when they are placed in any situation in which they are expected to mingle with other people, including people they already know, they get worried and anxious.
4. Staying calm during a competitive situation	Allen stays calm during his figure skating performance in international competition; he lands all of his difficult jumps perfectly while under tremendous pressure to do well on behalf of his country.	Some people stay calm during a competitive situation; they are able to perform well despite being under a considerable amount of pressure to live up to the expectations of others and themselves.
5. Having difficulty focusing on tasks for a long time	Raymond has difficulty focusing on writing the sales presentations required by his job; he repeatedly stops working to chat with co-workers, shop online, and watch viral YouTube videos.	Some people have difficulty focusing on tasks for a long time; their attention wanders and they engage in alternative activities that do not advance their work on the task at hand.
6. Drinking too much	Martin frequently drinks too many tequila shots; he knows that his system can really only handle one per hour, but always drinks at least three times that amount, vomits, and then has terrible hangovers the next day.	Some people drink too much; they knowingly ingest more alcohol than their digestive systems can adequately process in a short span of time, and do so more frequently than is advisable for maximum wellbeing.
7. Tending to be optimistic about the future	Sharon tends to be optimistic about her career trajectory; she anticipates that her own performance will be excellent and expects to get good job assignments and eventual promotions.	Some people tend to be optimistic about the future; they approach the world with positive expectations about what events will happen in the future and how those events will unfold.
8. Being very driven to achieve	Thomas is very intent on becoming a top executive at his corporation; he works 18-h days and has never missed a work meeting, although he has missed many of his children’s sports games and recitals.	Some people tend to be very driven to achieve; this involves putting the vast majority of their time, effort, and mental focus on achieving their goals and paying relatively less attention to other areas of life.

For each behavior, we developed an abstract version by describing the behavior as being common to a group of people. Each abstract version started with “Some people…” and described the behavior as generally applied to them without presenting any idiosyncratic variations. For the corresponding concrete version, we specified a person with a first name and instantiated the behaviors in the context of that particular person using concrete terms. The two versions were roughly equated for length (see Table 1).

As for Experiment 1, we conducted a pretest of these items to determine whether the abstract and concrete versions of each behavior were perceived to correspond to each other as intended. We recruited a separate group of 41 participants for this pretest, of whom five were excluded for failing the attention check. Of the remaining 36 pretest participants, 18 judged whether the abstract version was “a good abstract description” of the concrete version on a scale of 1–9 (where 1 = a very poor description; 9 = a very good description), yielding a mean rating of 7.61 (*SD* = 0.26). A separate group of 18 participants judged whether the concrete version was “a good example” of the abstract version on a scale of 1–9 (where 1 = a very poor example; 9 = a very good example), yielding a mean rating of 7.99 (*SD* = 0.23). Mean ratings by behavior were all at least 7.33.

#### Procedure

The main experiment used the same measures as Experiments 1 and 2. The procedure was the same as Experiment 1, except that each participant made judgments for only half of the items in Table 2, in order to keep the length of the experiment reasonable. As in Experiment 1, the items were counterbalanced so that the abstract and concrete versions of the same item appeared in separate halves of the experiment.

### Results

**Fig. 4:**
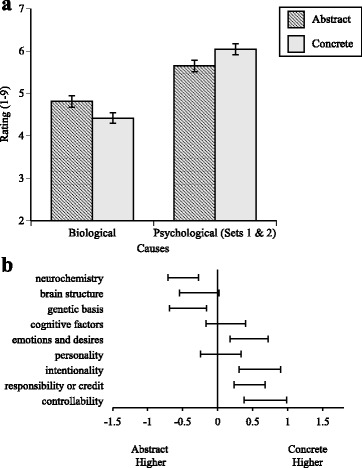
Results of Experiment 4. **a** Judgments of the biological and psychological bases of everyday behaviors rated within-subject; bars depict Cousineau–Morey standard errors (Cousineau, 2005; Morey, 2008). **b** The 95% confidence intervals of the difference scores (concrete minus abstract) for ratings on the nine dependent measures. “Biological Causes” in (**a**) are the averaged ratings of the first three dependent measures listed in (**b**), and “Psychological (Sets 1 & 2) Causes” are the averaged ratings of the last six dependent measures listed in (**b**)

Each participant’s biological (α = 0.95, calculated by item) and psychological (α = 0.85) attributions were averaged separately. We conducted a 2 (concrete or abstract) × 2 (biological or psychological) repeated measures ANOVA on each participant’s mean across items. This analysis revealed the predicted interaction, *F*(1,38) = 33.95, *p* < 0.001, η_p_^2^ = 0.47, as shown in Fig. 4a. Biological attributions were higher for the abstract versions (*M* = 4.81, *SD* = 1.22) than for the concrete versions (*M* = 4.42, *SD* = 1.12), *t*(38) = −4.36, *p* < 0.001, *d* = −0.70, while psychological attributions were higher for the concrete versions (*M* = 6.04, *SD* = 0.84) than for the abstract versions (*M* = 5.65, *SD* = 0.93), *t*(38) = 4.84, *p* < 0.001, *d* = 0.78.

As shown in Fig. 4b, the effects for each component measure were directionally consistent with our predictions and with previous experiments, but were somewhat more variable. Although six of the nine measures reached significance at the *p* < 0.05 level (two-tailed *t*-test against 0), one biological factor reached marginal significance (brain structure; *p* < 0.10), and two psychological factors did not significantly differ from 0 (cognitive factors and personality; see below for discussion).

The interaction effect also held up in a by-item analysis, using each item’s mean score across participants. A 2 (abstract or concrete) × 2 (biological or psychological) repeated measures ANOVA on these scores revealed the predicted interaction, *F*(1,7) = 16.62, *p* = 0.005, η_p_^2^ = 0.70. Biological attributions were higher for the abstract versions (*M* = 4.81, *SD* = 0.83) than for the concrete versions (*M* = 4.42, *SD* = 0.83), *t*(7) = −4.27, *p* = 0.004, *d* = −1.51, while psychological attributions were higher for the concrete versions (*M* = 6.04, *SD* = 0.58) than for the abstract versions (*M* = 5.65, *SD* = 0.90), *t*(7) = 2.65, *p* = 0.033, *d* = 0.94.

### Discussion

Experiment 4 found that shifts in attribution occur not only for mental disorders, but for a much broader range of human behaviors. These shifts were consistent across the three biological measures (albeit marginally significantly for brain structures), but somewhat more variable across the psychological measures. Although four of our psychological measures shifted significantly in the predicted direction, two others—cognitive factors and personality—did not.

Since all psychological measures shifted significantly in Experiments 1 and 2 depending on framing, it is worth considering why shifts were not seen for cognitive factors and personality in Experiment 4. We speculate that these somewhat less consistent effects of psychological attributions may be due in part to a weaker manipulation of abstractness that we used in Experiment 4, compared to Experiments 1–3. Whereas those previous experiments described the behaviors at the level of a category (a mental disorder) that did not invoke any individuals, Experiment 4 described the behaviors in terms of a group of individuals engaging in the behavior. Because even the abstract versions referred to human agents, they might have somewhat triggered psychological explanations. Furthermore, people may consider cognitive factors (e.g. beliefs and intelligence) and personality to be more immutable than the other, more transient psychological factors we tested, such as emotions and intentions. Nonetheless, significant shifts were still obtained for a majority of our measures of psychological attribution—and all measures of biological attribution (at least marginally significantly)—testifying to the robustness of the attributional shifts in the face of this weaker manipulation.

## Experiment 5

Experiment 5 sought to replicate the framing effects on attributions for ordinary behaviors, using a between-subjects design as in Experiment 2.

### Method

Two hundred and forty participants were recruited via Amazon Mechanical Turk, of whom 21 were excluded (*N* = 9 due to taking similar studies in the past and *N* = 12 due to random responses on filler items). Thus, data from 219 participants were used for the analyses.

The stimulus materials were the same as in Experiment 4. The design and the procedure were the same as in Experiment 2 in that participants received either the Biological (*N* = 36), the Psychological Set 1 (*N* = 145), or the Psychological Set 2 (*N* = 38) questions. Sample sizes were determined by power analyses on the data from Experiment 4, with 95% power subject to a minimum of 40 participants per condition (prior to excluding random responders and repeat participants).

### Results and discussion

**Fig. 5:**
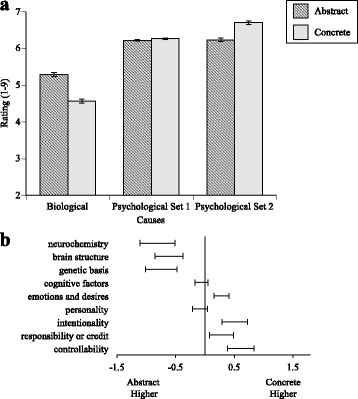
Results of Experiment 5. **a** Judgments of the biological and psychological bases of everyday behaviors rated between-subjects; bars depict Cousineau–Morey standard errors (Cousineau, 2005; Morey, 2008). **b** The 95% confidence intervals of the difference scores (concrete minus abstract) for ratings on the nine dependent measures. “Biological Causes” in (**a**) are the averaged ratings of the first three dependent measures listed in (**b**), “Psychological Set 1 Causes” the second three, and “Psychological Set 2 Causes” the last three

We conducted a 2 × 3 mixed-model ANOVA on each participant’s mean across items, with framing (concrete or abstract) as a within-subjects factor and attribution (Biological, Psychological Set 1, or Psychological Set 2) as a between-subjects factor. This analysis revealed the predicted interaction, *F*(1,228) = 51.15, *p* < 0.001, η_p_^2^ = 0.31, as shown in Fig. 5a. Biological attributions were higher for the abstract (*M* = 5.29, *SD* = 1.11) than for the concrete versions (*M* = 4.57, *SD* = 1.34), *t*(35) = −6.81, *p* < 0.001, *d* = −1.13, whereas the responses to the Psychological Set 2 questions were higher for the concrete (*M* = 6.71, *SD* = 0.74) than for the abstract versions (*M* = 6.24, *SD* = 0.95), *t*(37) = 5.16, *p* < 0.001, *d* = 0.84. The responses to the Psychological Set 1 questions did not differ between the concrete and abstract versions (*M* = 6.27, *SD* = 0.85 vs. *M* = 6.22, *SD* = 0.85), *t*(144) = 1.18, *p* = 0.24, *d* = 0.10, because cognitive abilities and personality—the two psychological measures that did not reach significance in Experiment 1—were unaffected by the manipulation. (See Fig. 5b for the 95% confidence intervals of the difference scores for each measure.) Again, we suspect that these less consistent effects on psychological attributions may be attributable to the weaker manipulation of abstractness used in Experiments 4 and 5, compared to Experiments 1–3, perhaps in conjunction with a tendency to view cognitive and personality factors as more immutable than the other psychological factors. Importantly, however, the effects on psychological attributions were significant overall and consistent for four of the six measures.

The interaction effect also held up in a by-item analysis. A 2 (abstract or concrete) × 2 (biological or psychological) repeated measures ANOVA on the item means revealed a significant interaction, *F*(1,7) = 38.80, *p* < 0.001, η_p_^2^ = 0.85. Biological attributions were higher for the abstract versions (*M* = 5.26, *SD* = 0.69) than for the concrete versions (*M* = 4.54, *SD* = 0.87), *t*(7) = −5.33, *p* = 0.001, *d* = −1.88, while psychological attributions were marginally higher for the concrete versions (*M* = 6.50, *SD* = 0.45) than for the abstract versions (*M* = 6.25, *SD* = 0.71), *t*(7) = 2.15, *p* = 0.069, *d* = 0.76. Follow-up analyses conducted separately on the two sets of psychological measures showed that this marginally significant effect on psychological attributions occurred because concrete items were rated significantly higher than abstract items on the Psychological Set 2 measures (*M* = 6.74, *SD* = 0.91 vs. *M* = 6.28, *SD* = 1.15), *t*(7) = 2.49, *p* = 0.041, *d* = 0.88, while the concrete and abstract items were rated similarly on the Psychological Set 1 measures (*M* = 6.27, *SD* = 0.33 vs. *M* = 6.22, *SD* = 0.45), *t*(7) = 0.59, *p* = 0.57, *d* = 0.21.

In sum, the results of Experiment 5 fully replicate the findings of Experiment 4, where biological attributions were consistently stronger in the abstract and psychological attributions were typically stronger in the concrete (with two of six measures failing to reach significance). Finding these same effects in a between-subjects design shows that the framing shifts cannot be due to a perceived demand to rate the psychological and biological explanations inversely.

## General discussion

In daily life, people often describe behaviors at differing levels of abstraction—as abstract generalizations across individuals or as concrete behaviors of individuals. We hypothesized that this distinction between abstract and concrete framing would lead to different explanatory preferences; namely, a stronger preference for biological explanations in the abstract and more reluctance to accept biological explanations for concrete cases.

The results across Experiments 1, 2, 4, and 5 corroborated this hypothesis. Both in contemplating disordered (Experiments 1 and 2) and everyday behaviors (Experiments 4 and 5), participants generally judged explanations in terms of genetics, neural chemistry, and brain structure to be more appropriate when faced with abstract descriptions of behavior than when faced with concrete cases. These differing explanatory stances also had downstream consequences such that people preferred a more “biological” intervention (medication) for treating disorders when described abstractly than when described in terms of a concrete case (Experiment 3).

It should also be noted that our claims are only about whether endorsement of biological and psychological explanations was influenced by abstract descriptions relative to concrete descriptions. Thus, we are not claiming that abstract framing would increase endorsement of biological explanations to the extent that they would be preferred to psychological explanations. In fact, this was not the case in Experiments 3–5. Similarly, we are not claiming that concrete framing would make psychological explanations be endorsed more than biological explanations; again, the current results failed to show that consistently (Experiments 1 and 2). Preferences for biological versus psychological explanations can vary greatly simply due to the nature of the events. For instance, “Don is full of himself” would be difficult to explain in terms of biological factors and thus although an abstract framing like “Some people are full of themselves” may make biological accounts more plausible, psychological accounts may still be more dominant than biological accounts even in the abstract framing.

In addition, we acknowledge that other factors may influence the availability of biological versus psychological explanations, including individual differences in theory of mind (Baron-Cohen, 1997), cognitive reflectiveness (Frederick, 2005), or even a desire to blame others for their behavior (Clark et al., 2014). We do not mean to downplay the importance of other potential factors, but rather seek to argue that the abstract/concrete distinction plays a key role.

### Possible mechanisms

In the introduction, we briefly presented two explanations for this framing effect. First, abstract framing, which conveys general patterns, triggers the need for more immutable explanations (e.g. Cimpian & Salomon, 2014), and biological properties are judged to be immutable and timeless (e.g. Dar-Nimrod & Heine, 2011; Lebowitz et al., 2013) just like generic abstract framing. Second, previous studies found that people more strongly attribute behaviors to free will when the events are described in more concrete contexts (e.g. Nichols & Knobe, 2007). We acknowledge that there are also other possible mechanisms for this framing effect and we briefly discuss three here: an inverse relationship between psychological and biological judgments, dualist thinking, and the influence of formal education.

#### Inverse relationship between psychological and biological judgments

People have been shown to behave as though biological and psychological explanations have an inverse relationship. That is, people sometimes behave as though factors making one kind of explanation more plausible correspondingly make the other kind less plausible (e.g. Preston, Ritter, & Hepler, 2013; see also Ahn, Proctor, & Flanagan, 2009 for similar findings with clinicians). Thus, salient psychological explanations for concrete cases may additionally suppress biological explanations and salient biological explanations for abstract cases may also additionally suppress psychological explanations. In that sense, this belief in an inverse relationship is not by itself an explanation for our effects because there should be an initial mechanism for making biological explanations salient for abstract cases or psychological explanations salient for concrete cases. Yet, once biological explanations become salient for abstract framing (due to, for instance, biological explanations being compatible with generic abstract framing), it may make psychological explanations less salient for abstract framing.

#### Dualist thinking

Recent work has explored the possibility that people are intuitive mind–body dualists, who believe that the mind and brain are separate entities (e.g. Bloom, 2007; Forstmann, Burgmer, & Mussweiler, 2012; Hood, Gjersoe, & Bloom, 2012; Hook & Farah, 2013). Whereas philosophers of mind hold that biology and psychology represent separable levels of analysis, such explanations are usually seen as complementary (e.g. Dennett, 1971). Laypeople may instead see these explanations as competing (e.g. Preston et al., 2013)—a form of dualism that is not inconsistent with the current findings.

The current results could also help to explain previous framing effects in judgments of free will. Nichols and Knobe (2007) found that people often endorse determinism in the abstract, but are more inclined toward belief in free will for individuals (Nichols & Knobe, 2007). Our results suggest one possible explanation for this result—that people are dualists in the sense that they do not juxtapose biological and psychological explanations, but rather treat them as competing explanations, privileging one over the other depending on the context. Our findings suggest that people may be subtly drawn to physicalism, the claim that everything is physical or is necessitated by the physical, more strongly in the abstract than in the concrete.

That said, our results do not present any direct demonstrations of Cartesian dualism, the claim that mind and body are distinct substances. We collected participants’ dualists beliefs at the end of Experiments 1 and 4, presenting them with the dualism scale from Stanovich (1989), and found that the framing effects did not correlate with people’s dualist beliefs. In this scale, participants judged their agreement with 27 statements (e.g. “the mind and the brain are two totally separate things;” “in a hundred years or more, it might make sense to refer to a computer as having a mind”) on a 5-point scale. For each participant, we computed the correlation between their scores on this dualism scale and the extent to which they showed the framing effect. As an index of framing effects, we added each participant’s difference score (i.e. concrete minus abstract) for psychological attributions to the opposite sign difference score (i.e. abstract minus concrete) for biological attributions. This provides an estimate of the interactive effect of concreteness/abstractness on psychological and biological attributions for each participant. The average correlation between the dualism scale and the framing effect was significantly negative in Experiment 1, *r*(41) = −0.38, *p* = 0.013, and failed to reach significance in Experiment 3, *r*(37) = 0.34, *p* = 0.16. Taken together, these findings speak against the possibility that those who are more likely to endorse mind–body dualism are more likely to be subject to the abstract/concrete framing effect. Nonetheless, these null results should be taken with caution, in part because the dualism scale may have become a less valid measure of dualist beliefs in recent years. That is, the pervasiveness of biological accounts of human behaviors may have made laypeople deny mind–body dualism when confronted explicitly, as is the case in the dualism scale. Future research, using more implicit measures of dualism, can help us better understand the shape and the scope of dualist beliefs that laypeople hold.

#### Context-sensitivity of intuitive and formal theories

People hold lay theories across many domains that differ dramatically from more formal scientific theories, including theories in biology (Shtulman, 2006), physics (McCloskey, 1983), statistics (Tversky & Kahneman, 1971), economics (Furnham & Argyle, 1998), personality (Haslam et al., 2004), decision theory (Johnson & Rips, 2015), and emotion (Gilbert & Wilson, 2007). Further, these lay theories often coexist in an individual’s mind with their formal counterparts (Shtulman & Valcarcel, 2012). Adults who have had many years of formal education and who would have no difficulty endorsing the appropriate scientific theory if asked explicitly nonetheless show slower response times in verifying facts that have different truth values on their formal and intuitive theories (e.g. “fire is composed of matter” or “air is composed of matter”), compared to facts that have the same truth values on both theories (e.g. “rocks are composed of matter” or “numbers are composed of matter”). Indeed, under time pressure, expert biologists fall back on their intuitive theories of biology, according to which plants are non-living (Goldberg & Thompson-Schill, 2009) and expert physical scientists endorse teleological explanations for physical phenomena (e.g. “Trees produce oxygen so that animals can breathe”; Kelemen, Rottman, & Seston, 2013).

Very little is known, however, about what circumstances lead individuals to apply their formal versus intuitive theories to a problem when these theories disagree. We speculate that people may be more likely to rely on their formal theories in the abstract and more likely to default to their earlier, intuitive theories in the concrete. This idea can provide a further mechanism for the current findings. Whereas folk psychology is a natural and early-emerging mode of explanation (e.g. Gergely & Csibra, 2003; Onishi & Baillargeon, 2005), brain-based biological explanations seem to emerge later (Johnson & Wellman, 1982). Further, people usually learn about biological explanations in an abstract format. For example, science-based websites for the public that explain the biological underpinnings of behavioral disorders (e.g. from such authoritative bodies as the CDC, NIH, and Mayo Clinic) invariably describe what is known about each disorder in general, rather than describing individual case studies. Student textbooks explaining the biology of behaviors and commercials marketing psychotropic medications often take the same approach. Consequently, formally acquired biological explanations for behavior may seem relatively natural in the abstract, but people may default to their lay theories such as folk psychology in the concrete, accounting for our framing effect.

One way to test the formal education hypothesis would be to ask whether an analogous effect arises in other domains. Would people apply different lay economic theories in contemplating one individual country versus countries in general? Would people apply different lay theories of evolution in contemplating one particular species versus species in general? Would people give different advice about how to maximize happiness if the advice is applied to a particular person versus people in general? To the extent that formal and intuitive theories may give different verdicts, these questions may be of considerable practical importance.

A second way to test the hypothesis would be to conduct developmental studies. Presumably, young children do not have a formal education in biology or neuroscience, so if the effect is indeed driven by formal education, it should not arise among young children. By contrast, if the effect is driven by an intuition that biological explanations are tied to immutability and hence essentialism, it might arise much earlier in development. For instance, Cimpian and Markman (2011) found that when asked to explain either generic statements (e.g. boys are good at math) or non-generic statements (e.g. Johnny is good at math), even four-year-olds preferred to explain generic statements in terms of inherent features (e.g. “because that’s how they’re made”) than extrinsic features (e.g. “because they got teached”). This effect of genericity on intuitions about inherence does not seem to require formal education, and if our framing effects are driven by the same process, they might be similarly early-emerging. On the other hand, our results are more nuanced in that people distinguished between biological explanations and psychological explanations, when both (or at least some of the psychological explanations used in the current study) are treated as inherent and essentialized explanations in the previous developmental studies. This finer distinction may emerge later in development as a result of learning biological theories in the abstract context.

### Implications for Public Health and Science Education

We found that, like clinicians (Kim et al., 2016), laypeople endorse different explanations for mental disorders in the abstract and in the concrete (Experiments 1 and 2), which can even lead to different treatment recommendations (Experiment 3). These results have implications for public communication about mental disorders. Biological explanations of psychopathology lead people to essentialize mental disorders (e.g. Dar-Nimrod & Heine, 2011; Haslam & Ernst, 2002), to distance themselves from or reduce empathy toward people who have mental disorders (Lebowitz & Ahn, 2014; Read, Haslam, Sayce, & Davies, 2006), and to be more pessimistic about mental disorder prognoses (Deacon & Baird, 2009; Kvaale, Haslam, & Gottdiener, 2013). At the same time, however, these explanations can ameliorate stigma by reducing personal blame for mental disorder symptoms (e.g. Deacon & Baird, 2009). These studies, along with the current results, suggest that, depending on the goal of communication, it may be best to use either abstract or concrete descriptions. One should use concrete descriptions if one wishes to de-essentialize mental illness or improve perceived prognosis and abstract descriptions if one wishes to reduce blame for the symptoms.

Our finding also has implications for science education more broadly. Science educators have long debated the relative value of abstract and concrete teaching materials (see Fyfe, McNeil, Son, & Goldstone, 2014 for a review). Concrete materials have both advantages (e.g. they may be more likely to utilize real-world knowledge; Schliemann & Carraher, 2002) and disadvantages (e.g. they can also distract with extraneous perceptual details; Belenky & Schalk, 2014); yet abstract materials, too, have their own benefits (e.g. they emphasize structural features over superficial features; Uttal, O’Doherty, Newland, Hand, & DeLoache, 2009) and pitfalls (e.g. mindless symbol manipulation; Nathan, 2012). It is often noted that because of these complementary advantages and disadvantages, the use of both kinds of materials is necessary. However, our results suggest another critical difference between these types of materials—whereas the use of biological explanations (acquired through science education) may be relatively natural in an abstract setting, students may fall back on their psychological explanations in concrete settings. This highlights the need, not only to expose students to both kinds of teaching materials, but to map the connections between concrete problems and their abstract logical structure, if educators hope for the biological explanations they are teaching to their students to be generalized to the concrete world.

## Conclusion

We explain human behaviors in multiple ways. We can emphasize the importance of responsibility, controllability, intentions, beliefs, and desires. We can also explain human behavior in terms of biological forces, such as genes, neural chemistry, and brain structure. Our results showed that biological theories of behavior are more privileged when contemplating abstract descriptions rather than concrete cases. Thus, even though abstract and concrete descriptions of behavior are both ubiquitous in the world, and often seemingly equivalent, they can nonetheless lead to very different inferences about the causes underlying the behavior.

## Footnotes

1 Although the *DSM-5* (American Psychiatric Association, 2013) is the most recent version of the manual, *DSM-IV-TR* (American Psychiatric Association, 2000) was the only version available at the time we developed these materials. Nevertheless, any statements made in this paper in reference to the *DSM-IV-TR* are also valid in terms of the *DSM-5*, as the particular symptoms we used remain in the *DSM-5*.
